# Maritime Emission Monitoring: Development and Testing of a UAV-Based Real-Time Wind Sensing Mission Planner Module

**DOI:** 10.3390/s24030950

**Published:** 2024-02-01

**Authors:** Theodoros Karachalios, Panagiotis Moschos, Theofanis Orphanoudakis

**Affiliations:** 1 Digital Systems and Media Computing Lab, School of Sciences and Technology, Hellenic Open University, 263 34 Patra, Greece; panagmosx@hotmail.com (P.M.); fanis@eap.gr (T.O.); 2 Department of Industrial Design and Production Engineering, University of West Attica, 122 41 Egaleo, Greece

**Keywords:** UAV, environmental monitoring, maritime emission tracking, ArduPilot mission planner, real-time data analysis, dynamic path planning, air quality assessment, pollution control technologies, emission dispersion modeling

## Abstract

Maritime emissions contribute significantly to global pollution, necessitating accurate and efficient monitoring methods. Traditional methods for tracking ship emissions often face limitations in real-time data accuracy, with wind measurement being a critical yet challenging aspect. This paper introduces an innovative mission planner module for unmanned aerial vehicles (UAVs) that leverages onboard wind sensing capabilities to enhance maritime emission monitoring. The module’s primary objective is to assist operators in making informed decisions by providing real-time wind data overlays, thus optimizing flight paths and data collection efficiency. Our experimental setup involves the testing of the module in simulated maritime environments, demonstrating its efficacy in varying wind conditions. The real-time wind data overlays provided by the module enable UAV operators to adjust their flight paths dynamically, reducing unnecessary power expenditure and mitigating the risks associated with low-battery scenarios, especially in challenging maritime conditions. This paper presents the implementation of real-time wind data overlays on an open-source state-of-the-art mission planner as a C# plugin that is seamlessly integrated into the user interface. The factors that affect performance, in terms of communication overheads and real-time operation, are identified and discussed. The operation of the module is evaluated in terms of functional integration and real-time visual representation of wind measurements, and the enhanced situational awareness that it can offer to mission controllers is demonstrated. Beyond presenting a novel application of UAV technology in environmental monitoring, we also provide an extensive discussion of how this work will be extended in the context of complete aerial environmental inspection missions and the future directions in research within the field that can potentially lead to the modernization of maritime emission monitoring practices.

## 1. Introduction

Maritime emission pollution poses a significant threat to both environmental and human health, contributing to global issues such as climate change, ocean acidification, and air quality degradation. Ships navigating the oceans have achieved relatively low fuel consumption per unit of cargo distance, yet they emit significant amounts of pollutants, including sulfur oxides (SOx), nitrogen oxides (NOx), and particulate matter (PM). These emissions have considerable impacts on both the atmosphere and marine ecosystems. Near coastal areas, maritime emissions are subject to stringent regulations, such as the requirement to use low-sulfur fuel. This makes the monitoring of emissions near shorelines crucial to ensure compliance with these regulations and to mitigate the harmful effects of these pollutants. The importance of closely observing and regulating these emissions in coastal areas is a key component in efforts to protect the environment [1,2,3].

Addressing concerns about air pollution from shipping emissions and observing its upward trend, the International Maritime Organization has set a series of standards and guidelines for the measurement, analysis, and documentation of sulfur emissions from ships [4]. The main purpose is the reduction of environmental and health impacts from air pollution caused by shipping. Central to these efforts is the limitation of the sulfur content of fuel oil, with particular emphasis on emission control areas (ECAs)—zones in northern Europe and North America known for heavy shipping traffic. These regulations cover more than 30% of the air pollution concerns and require ships to run on low-sulfur fuels. In addition, from 2021, ships in the northern and Baltic ECA and northern American ECA must comply with Tier III NOx standards. Especially for liquid fuel tanks, the main strategy for managing emissions includes the use of selective catalytic reduction (SCR) units. It is critical to ensure the effectiveness of these units within the EACs, and for this reason, additional monitoring is deemed necessary. Ships are not only sources of sulfur oxides (SOx) and nitrogen oxides (NOx), but they also emit a range of particulates, including black carbon (BC), organic carbon (OC), ash, and metal aerosols, mainly in the ultra-fine particle (UFP < 100 nm) range. As regulations on particulate emissions are not currently regulated, particularly regarding BC due to its significant climate impacts, increasingly, people are realizing the importance of implementing more detailed and effective strategies for management and oversight. In addition, aerosols, through the photooxidation of SOx, NOx, and OC, impact the coastal environment significantly. The EU has taken additional measures, implementing regulations more strictly for ferries and ships at anchor that go beyond the scope of IMO standards. These regulations require discrete monitoring for sulfur compliance both at sea and in port areas.

**Figure 1:**
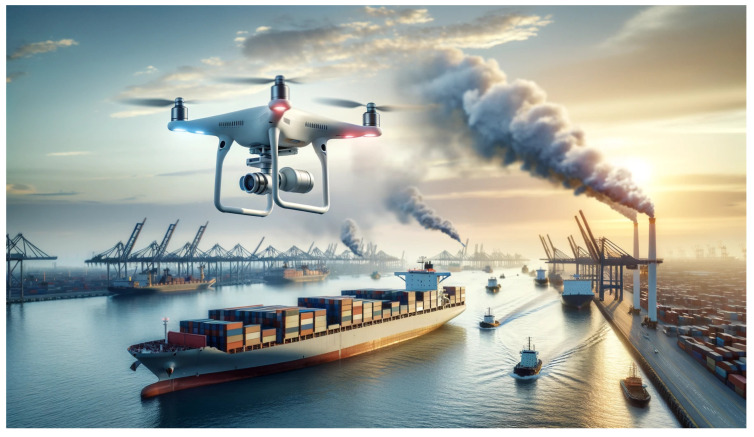
Artistic representation of maritime emission monitoring mission.

However, the implementation of these regulations faces challenges, mainly due to the higher cost of low-sulfur fuels, which cost almost twice as much as high-sulfur alternatives. This is the main reason that creates a disincentive for the shipping industry to comply. In response, national authorities such as environmental and maritime agencies have recently begun to develop remote monitoring technologies to monitor ship emissions in environmental zones (Figure 1). This enforcement strategy serves as a deterrent and documentation of non-compliance, requiring action by local authorities. Fuel sampling at ports is a smart targeting approach using air sampling, which optimizes port inspections. Current solutions for remote monitoring of sulfur emissions include fixed measurement stations that analyze exhaust plumes from passing ships and aerial surveillance, using gas analyzers mounted on helicopters, airplanes, or drones. Airborne surveillance offers advantages over fixed stations because it allows the aircraft to navigate close to any exhaust cloud condensation for better quality air sampling and covers larger areas, conduction unexpected inspections to improve compliance enforcement. The above facts underpin the importance of accurate and reliable monitoring of maritime emissions for enforcing environmental regulations and guiding policy decisions. However, the efficacy of aerial monitoring is inherently tied to understanding and tracking the dispersion of pollutant plumes, which are significantly influenced by wind patterns. Traditionally, emission monitoring systems have relied on predictive wind models to estimate plume trajectories. These models, while useful, often fall short of capturing the dynamic and complex nature of maritime wind patterns [5,6], leading to potential inaccuracies in emission tracking and assessment [7].

The dynamic nature of the maritime environment presents unique challenges, where wind conditions can change rapidly and unpredictably [8], affecting the dispersion and concentration of pollutants. The reliance on wind predictions, as opposed to real-time wind data, introduces a notable gap in the accuracy and responsiveness of current monitoring methodologies. Recognizing this, our research introduces an innovative approach that integrates real-time wind sensing into UAV operations. This method not only offers a more immediate and precise understanding of wind patterns but also significantly enhances the UAVs’ ability to adapt their mission plans accordingly, ensuring more accurate and safer [9] emission monitoring.

By shifting the focus from predictive [10] to real-time data, our approach addresses a critical need in maritime emission monitoring. It represents a significant step forward in environmental monitoring technology, paving the way for more effective and responsive strategies in combating maritime pollution. This paper details the development and application of this novel UAV-based mission planner module, highlighting its potential to revolutionize the field of maritime emission monitoring.

In this study, we introduce a pioneering approach to UAV-based maritime emissions monitoring by integrating real-time wind sensing into the mission planning process. This novel development is not just an incremental improvement but a significant leap forward in the field of UAV navigation and environmental monitoring. By harnessing the power of real-time wind data, our system enhances the precision and effectiveness of UAVs in challenging maritime environments. This paper details the innovative aspects of our approach, from the integration of wind overlays in mission planning to the dynamic adjustment of UAV flight paths based on wind patterns. The result is a more intelligent, responsive, and efficient system, tailored specifically for the complex demands of maritime emission monitoring. Our work stands out for its practical application of advanced UAV technology in environmental research, marking a notable contribution to both the field of unmanned aerial systems and environmental monitoring.

The remainder of this manuscript is organized as follows: in Section 2, we review related published research articles to focus our work on the current state of the art for implementing aerial environmental inspection missions and highlight, on the one hand, the importance of efficient real-time wind measurements, and on the other hand, the lack of opensource solutions towards this end; in Section 3, we describe the theoretical framework and background supporting our implementation; in Section 4, we present the methodology for integrating the real-time wind measurement and visualization component into the mission planner software; in Section 5, we present the module design and in Section 6, we present the results of the module operation in a testing and verification environment; in Section 7, we discuss the next steps towards extending our application for environmental inspection mission planning, in conjunction with addressing some challenges and limiting factors that are discussed in Section 8; finally, Section 9 provides the main conclusions of our work.

## 2. Literature Review

The application of unmanned aerial vehicles (UAVs) in wind data collection [11,12,13], particularly in the atmospheric boundary layer, marks a significant advancement in environmental monitoring technologies. UAVs, both fixed-wing and rotary-wing, equipped with various anemometer technologies, have been instrumental in enhancing short-term wind speed measurements.

UAV-based wind speed measurements can be categorized into indirect methods, which measure the UAV’s response to wind [14], and direct methods, which use dedicated wind sensors [15]. Indirect methods require understanding the UAV’s inertia and drag coefficients, while direct methods involve the use of lightweight, robust 3D wind sensors. The fusion of vehicle speed and wind speed is crucial in direct methods but can yield errors due to inaccuracies in sensing and state estimation.

Different types of sensors, such as differential pressure sensors and hot-wire anemometers, have been used for UAV-based wind speed measurements. Each sensor type has its advantages and limitations. For example, differential pressure sensors such as pitot tubes are suitable for larger area coverage, while hot-wire anemometers offer high-frequency measurements but are fragile. Sonic anemometers provide accurate measurements but face challenges with size and weight constraints.

The use of miniature sonic sensors [16] on UAVs is increasing, offering the potential for accurate 3D wind measurements. However, achieving accurate measurements with these sensors can be challenging due to factors such as shadowing effects, especially when the UAV is not flying perfectly leveled. Careful calibration and validation [17,18] of these sensors are essential for proper wind readings.

## 3. Theoretical Framework

The theoretical underpinnings of maritime emission monitoring are rooted in environmental science and atmospheric chemistry, particularly in understanding the behavior and impact of pollutants released by maritime vessels. Central to this is the study of pollutant dispersion in the atmosphere, a process heavily influenced by meteorological conditions, primarily wind speed and direction [19]. The dispersion of these emissions follows complex dynamics, governed by both atmospheric stability and the turbulent mixing of air masses. This complexity necessitates a multi-disciplinary approach, combining principles from fluid dynamics, thermodynamics, and environmental chemistry. Traditional models for predicting pollutant dispersion rely on historical meteorological data and theoretical wind patterns. While these models provide a foundational framework for understanding emission dispersion, they often lack the granularity and adaptability required to capture the rapidly changing conditions in a maritime context [20]. This gap underscores the need for a more dynamic and responsive approach to emission monitoring.

The emergence of UAVs with an adequate mission range, capable of carrying a payload that facilitates efficient sensing, data logging, and communications offers an agile and sophisticated platform for environmental monitoring. The integration of UAVs with real-time wind sensing technology marks a significant evolution in monitoring capabilities. This approach is grounded in the theory of adaptive environmental sensing, where data collection is responsive to environmental conditions, allowing for more accurate and timely assessments. Real-time wind sensing on UAVs involves the application of aerodynamic principles [21] and advanced sensor technology to measure and interpret wind velocities and directions instantaneously. These data not only enrich our understanding of emission dispersion patterns but also enable the UAVs to adjust their flight paths dynamically using novel algorithms [22], optimizing the collection of environmental data. The theoretical framework of this approach is a combination of UAV technology, atmospheric science, and data analytics, working in concert to provide a more precise and reliable method for maritime emission monitoring. By leveraging real-time data, this approach promises a significant advancement in our capacity to monitor and mitigate the impacts of maritime emissions, providing accurate monitoring through UAV-based systems. Moreover, the UAV task planning module is tailored for UAV categories 1, 2, and 3, which typically operate within telemetry range, well within their flight range limits. This ensures reliable communication throughout their operation. Importantly, wind data are already integrated into the MAVLink messages, adding no extra communication overhead. In addition, all necessary calculations are handled by the host machine, avoiding additional processing load on the flight module itself. This design effectively balances operational range, communication efficiency, and processing power for optimal UAV performance.

## 4. Methodology

Our methodology for developing the mission planner module for ArduPilot-powered UAVs in maritime emission monitoring is rooted in the innovative application of existing embedded algorithms from ArduPilot/ardupilot firmware, specifically sourced from ArduPlane, ArduCopter, ArduRover, and ArduSub on GitHub (github.com, accessed on 1 January 2024). The primary focus is not on creating new algorithms but on creating a novel method of overlaying embedded algorithms readings to enhance the UAV’s operator mission planning capabilities. The key development in this module is the mechanism for extracting wind data from onboard sensors [23], which are then carried into MAVLink messages, and effectively integrate this information into the UAV’s mission planning graphical user interface (GUI). The integration enhances situational awareness for drone operators by offering a rapid recognition of wind conditions. This feature allows operators to dynamically adjust the drone’s flight path for more accurate readings and enhanced safety, as opposing winds could result in early landing failsafe triggers due to low battery [24]. Visual identification of opposing winds is particularly beneficial, as it helps in conserving battery power by avoiding routes with strong headwinds during the return journey for landing. This system not only contributes to the precision of emission data collection but also significantly increases the safety and efficiency of drone operations.

To test and validate the C# code of the mission planner module, the study employs the SITL simulator [25], a sophisticated tool that allows for comprehensive and controlled testing of UAV behavior in simulated environments. This simulation approach enables the precise programming of various wind parameters, creating diverse scenarios to assess the module’s responsiveness to variable wind conditions. The testing process involves simulating different maritime emission scenarios, where the UAV, equipped with the mission planner module, is required to navigate and display data based on the simulated wind patterns.

**Figure 2:**
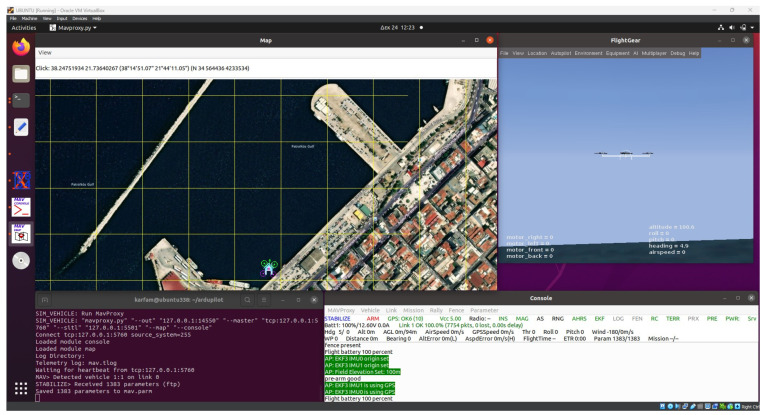
SITL running in Ubuntu VM.

This simulated testing environment provides (Figure 2) a safe and efficient means to evaluate the module’s performance and the effectiveness of its algorithm while travelling through the box grid.

## 5. Module Design

### 5.1. Module Design and Integration

The development of the mission planner module is carried out using Visual Studio 2022, utilizing C# for its robust capabilities in handling complex data processing and user interface development. This choice ensures a high degree of compatibility and performance when integrated with the mission planner software, a critical aspect considering the software’s widespread use in UAV operations. The module’s architecture is tailored to fit seamlessly within the mission planner’s existing framework, enabling a smooth compilation process. This integration transforms the module into a vital component of the UAV’s control system, seamlessly integrating with the software’s existing features and introducing new functionalities. While it is specifically advantageous for maritime emission monitoring missions, it is also a significant upgrade for all users of ArduPilot firmware seeking detailed insights into crucial flight aspects like wind conditions. By providing more nuanced information, it enhances overall flight control and decision-making, making it a valuable tool for a wide range of UAV applications.

### 5.2. Functionality and Real-Time Data Processing

**Figure 3:**
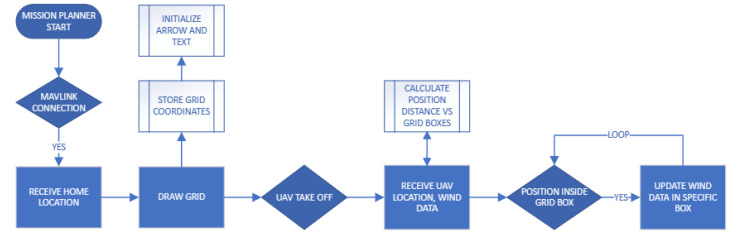
Module logic flow chart.

The module’s operational process (Figure 3), initiates with the reception of MAVLink [26] messages, a standardized communication protocol in UAV systems, which provide essential information, such as the UAV’s current location. Data transmission between UAV and the ground system is facilitated through telemetry modules such as SIK radio. This process is described in detail on the telemetry landing page of the copter documentation on ardupilot.org, https://ardupilot.org/copter/docs/common-telemetry-landingpage.html, accessed on 1 January 2024. Furthermore, the encapsulation of these data is efficiently executed using the MAVLink protocol. The mission planner module in our UAV system is adept at handling real-time data by using an event listener mechanism that is attuned to MAVLink messages. MAVLink, being the communication protocol for drones, plays a critical role in transmitting vital data, including wind measurements. MAVLink message is configurable and can operate at a frequency ranging from 1 to 50 Hz, depending on the settings chosen. As the UAV operates, it continuously receives MAVLink messages containing wind measurements. These messages are crucial for the real-time functionality of the module. Each message is processed as it arrives, and the contained wind data are immediately utilized for overlay drawing on the mission planner interface.

**Figure 4:**
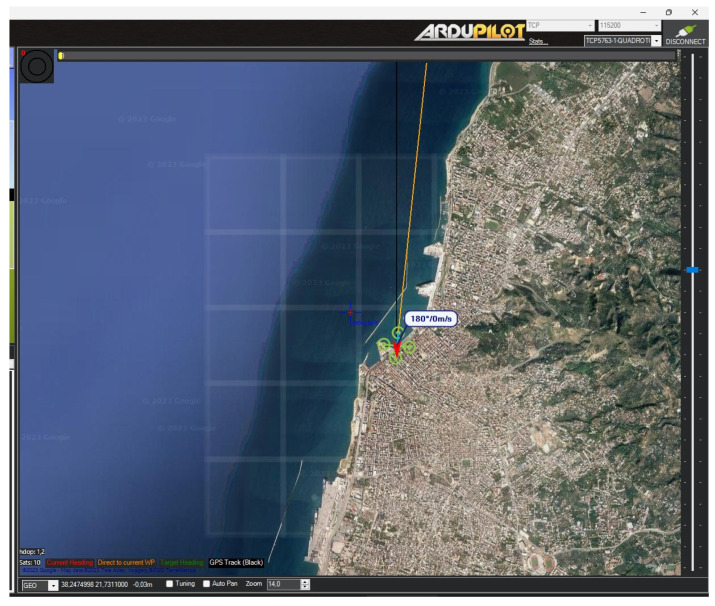
Initial grid overlay during the initialization of the module.

Upon receiving initial data (Figure 4), the module activates a particularly useful feature: it generates a custom grid overlay on the mission map. This grid is specifically designed to encompass a 1000 m × 1000 m area, divided into 25 equal squares in a 5 × 5 layout. The size of the grid, covering a 5 km range, aligns with the most common UAV range specifications, making it broadly applicable and useful. This structured spatial representation around the UAV enhances navigation precision and situational awareness, proving invaluable for strategic planning and decision-making in various UAV operations. Each square in the grid is equipped with two dynamic elements: an arrow indicating the wind direction and a text label that shows the wind speed and direction. These elements are not static; they are programmed to become visible only when there is a measurement taken by the drone in that specific area. This feature ensures that the overlays are not only relevant and informative but also unobtrusive, displaying crucial information exactly when and where it is needed. By focusing on areas where the drone has collected data, the system enhances the efficiency of the data presentation, allowing operators to quickly identify and focus on key regions of interest. This tailored visibility approach helps in maintaining a clean and clear mission map, free from unnecessary clutter, thereby aiding in better decision-making and more precise navigation.

### 5.3. Adaptive Environmental Monitoring

As the UAV takes off and commences its mission, the module’s functionality expands. It consistently monitors the UAV’s position and the incoming wind data, adjusting the graphical representation of the latest measurements in terms of wind direction and speed. This dynamic updating is critical in maintaining the relevance and accuracy of the environmental data displayed on the mission map. When the UAV navigates into a specific grid square, the algorithm undertakes a crucial task: it calculates the average wind direction and speed based on the data collected within that area. This calculation ensures that the environmental data for each specific grid square is as accurate and representative as possible. The module’s focus on localized and real-time environmental data processing is a substantial advancement in UAV mission control technology, applicable not only to maritime emission monitoring but also for everyday usage. By providing operators with precise, real-time environmental data, the module significantly enhances situational awareness and interaction with the environment. By enabling operators to make informed decisions about flight paths in response to changing wind conditions, the UAV can operate more efficiently, reducing unnecessary battery consumption. This not only extends the UAV’s operational range but also ensures a safer return to base, crucial in all UAV applications.

### 5.4. Dynamic GUI Elements for Enhanced Visualization

**Figure 5:**
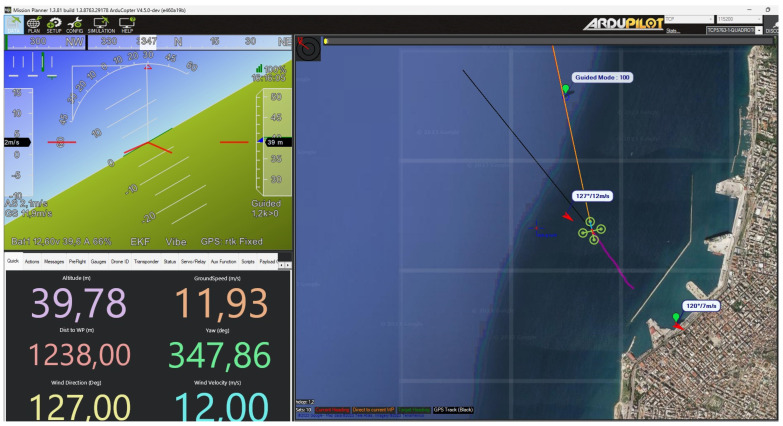
Grid wind data update after UAV measurement.

One of the key enhancements in the GUI of the mission planner module is the dynamic representation of wind speed (Figure 5). This is achieved through the innovative use of size-changing arrows and color-changing text labels within each grid box. The arrow indicating wind direction varies in size proportionally to the wind speed: the stronger the wind, the larger the arrow. This visual cue provides a quick and intuitive understanding of wind intensity, allowing UAV operators to assess conditions immediately. Similarly, the text label displaying wind speed and direction changes color based on predefined thresholds of wind intensity. For example, a shift from green to yellow to red could represent increasing wind speeds, providing a clear and immediate visual indicator of changing environmental conditions. These dynamic GUI elements are designed not only for aesthetic appeal but, more importantly, for enhancing situational awareness and aiding in quick decision-making during UAV missions.

## 6. Testing Environment

### 6.1. Testing Setup and Virtual Environment Configuration

**Figure 6:**
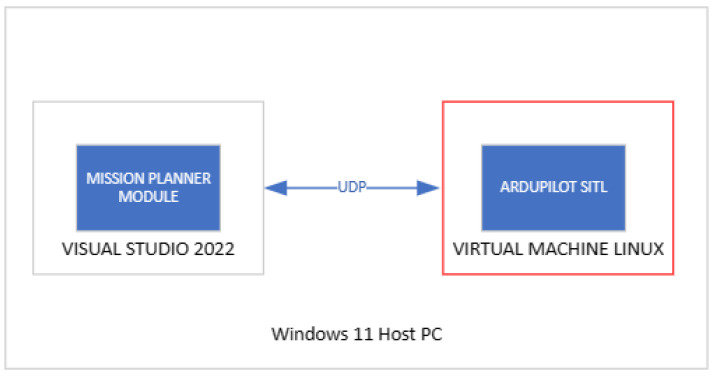
Setup of test environment.

The testing of the mission planner module begins with the meticulous configuration of a virtual environment (Figure 6), essential for simulating real-world UAV operations. This setup involves the integration of ArduPilot firmware with the SITL simulator, a key component in replicating UAV flight dynamics without the need for actual flight tests. The SITL simulator provides a controlled and flexible environment, enabling comprehensive testing of the UAV’s response to various scenarios and conditions. To connect the mission planner module developed in Visual Studio with the SITL simulator, a virtual machine is established, serving as a UDP bridge between the module and the simulator. This connection is facilitated through the Visual Studio emulator, which allows the plugin to communicate seamlessly with the SITL simulator, replicating the interaction that would occur between the UAV and the mission planner module in a real operational setting.

### 6.2. SITL Simulator Configuration for Wind Data Transmission

In the second phase of testing, the SITL simulator is specifically configured to emulate wind conditions and transmit MAVLink messages containing wind measurements. This configuration is crucial for validating the module’s ability to process and respond to real-time wind data, a fundamental aspect of its functionality. The simulator is set to generate a range of wind speeds and directions (Table 1), mimicking the variable conditions that are commonly encountered in maritime environments. This setup ensures that the module is tested under diverse scenarios, challenging its adaptability and robustness. The MAVLink messages sent from the simulator, carrying detailed wind information, are received by the mission planner module through the virtual machine setup.

This process tests the module’s capability to accurately interpret and utilize the wind data, a critical step in assessing the module’s real-world applicability. When conducting wind testing using the SITL simulator for the UAV-based mission planner module, it is crucial to configure certain parameters within ArduPilot firmware to ensure accurate simulation and data collection. The following settings are vital:

- BARO1_WCF_ENABLE: Set this parameter to 1. Enabling this setting is essential, as it activates specific barometer wind compensation features, allowing for more precise wind measurements and better simulation of real-world wind effects on the UAV.
- EK3_DRAG_BCOEF X, Y MCOEF: This copter documentation (ardupilot.org) provides guidance on calculating and assigning appropriate values, estimating windspeed, and compensating for baro pressure with respect to these coefficients. These parameters are part of the extended Kalman filter 3 (EKF3) settings, which handle the UAV’s drag estimation. Properly setting these coefficients is important for accurately simulating how wind affects the UAV’s movement.
- EK3_ENABLE: Set this to 1 to enable the EKF3 (extended Kalman filter version 3). EKF3 is more advanced and typically preferred for handling the UAV’s state estimation, which includes interpreting wind effects.
- EK2_ENABLE: Set this to 0 to disable EKF2, ensuring that the system relies solely on EKF3 for state estimation. Having both EKF2 and EKF3 active simultaneously could lead to conflicts or inaccuracies in data interpretation according to the firmware manual.
- AHRS_EKF_TYPE: Set this parameter to 3 to specify that the system should use EKF3. This setting ensures that all attitude and heading references are calculated based on the data processed by EKF3.

Moreover, importing specific wind parameters into the SITL simulator enables us to mimic a variety of maritime atmospheric conditions, ranging from calm breezes to challenging gusts. This level of simulation fidelity ensures that the module can reliably interpret and respond to diverse wind scenarios, a critical aspect for successful maritime emission monitoring. The specific parameters used in our simulation are detailed in Table 2.

### 6.3. Testing and GUI Response Evaluation

The final stage of testing focuses on the practical application and responsiveness of the mission planner module’s GUI. As the simulated UAV enters a specific grid box, the module is evaluated for its ability to update the GUI with the correct wind values. This test is vital in determining the module’s efficiency in real-time data processing and visualization. The GUI is designed to dynamically display an arrow indicating wind direction and a text label showing wind speed and direction, within each grid box. As the simulated UAV moves through different grid boxes, the module continuously updates these elements based on the latest wind data received. This test not only examines the accuracy of the wind data displayed but also assesses the module’s capacity to update the GUI promptly and accurately, in alignment with the UAV’s position within the grid. The success of these tests is indicative of the module’s potential effectiveness in real maritime emission monitoring scenarios, showcasing its capability to provide UAV operators with timely and accurate environmental data essential for informed decision-making.

## 7. Future Enhancements

### 7.1. Integration of Interactive Data Layers and Customization Options

A future enhancement for the GUI could involve the integration of interactive data layers, allowing operators to overlay additional environmental information onto the mission map. These layers could include real-time sea state data, weather forecasts, or even areas of ecological sensitivity. By providing a more holistic view of the maritime environment, operators can make more informed decisions regarding UAV flight paths and data collection strategies. Additionally, the introduction of customizable GUI themes and layouts would cater to individual preferences and operational requirements. This customization could include adjustable data display formats, such as graphs or gauges, and the ability to prioritize certain data types over others. These enhancements would not only improve the overall user experience but also increase the operational versatility of the module, making it adaptable to a wide range of maritime monitoring scenarios.

### 7.2. Advanced Analytical Tools and Collaborative Features

Looking further ahead, the GUI could be equipped with advanced analytical tools, such as predictive analytics algorithms that use historical data to forecast future wind patterns and emission dispersion trends. This feature would provide operators with predictive insights, aiding in strategic planning and risk assessment. Another innovative addition could be the implementation of collaborative features, enabling real-time sharing of UAV data and mission plans with other stakeholders, such as environmental agencies or maritime authorities. This collaboration could facilitate coordinated response efforts in environmental monitoring and emergency situations. The GUI could also incorporate augmented reality (AR) elements [27], providing an immersive experience where operators can visualize wind data and UAV paths in a three-dimensional space, further enhancing situational awareness and decision-making capabilities. Such enhancements would transform the GUI into a more powerful and interactive tool, significantly advancing the capabilities of UAV-based maritime emission monitoring and the situational awareness of UAV operators.

### 7.3. Automated Pre-Mission Wind Measurement Planning

A pivotal enhancement for the future development of the UAV-based mission planner module is the integration of an automated system for pre-mission wind measurement planning. This advanced feature would enable the module to autonomously plan and execute wind measurement missions prior to the primary maritime emission monitoring operation. By doing so, the UAV can collect comprehensive wind data across the intended area of operation, providing a detailed understanding of the prevailing wind conditions.

This automated pre-mission planning would involve the UAV systematically covering the target area, gathering wind speed and direction data at various altitudes and locations. This pre-collected data would be instrumental in creating a more accurate and detailed wind profile of the area, significantly enhancing the precision of subsequent emission monitoring missions. The module would use this data to optimize the flight path for emission monitoring, taking into account the most current and comprehensive wind information available.

Moreover, this capability would greatly improve operational efficiency. By automating the process of wind data collection, operators can focus on analyzing the data and planning the main emission monitoring mission more effectively. It also opens the door for more advanced predictive modeling, where the pre-collected wind data can be used to forecast wind patterns over the short term, further refining mission planning and execution. The implementation of automated pre-mission wind measurement planning represents a forward-thinking approach to UAV-based environmental monitoring. It not only enhances the current capabilities of the mission planner module but also aligns with the broader goals of utilizing technology for more efficient and accurate environmental assessment and management.

### 7.4. Customizable Grid Settings for Enhanced Flexibility

A significant enhancement planned for the future iteration of the UAV-based mission planner module is the introduction of a customizable settings box for grid configuration. This feature would allow operators to tailor the size of the grid box and the number of rows and lines according to the specific requirements of each maritime emission monitoring mission. By providing this level of customization, operators can optimize the grid layout for different environmental conditions and mission objectives. For instance, a larger grid with more rows and lines might be ideal for wide-area monitoring, while a smaller, denser grid could be better suited for detailed analysis in a confined area. The drawing of the grid could be automated and made more reliable based on wind thresholds. Larger boxes could be used for stable wind conditions, while smaller and more precise boxes could be employed for varying wind conditions. This flexibility in grid configuration is not just a convenience; it represents a significant step towards more adaptive and precise environmental monitoring. By enabling operators to adjust the grid parameters dynamically, the module will offer enhanced responsiveness to changing conditions, thereby improving the accuracy and efficiency of data collection and analysis in maritime emission monitoring missions.

### 7.5. Exporting Detailed 3D Graphs for Comprehensive Wind Data Analysis

**Figure 7:**
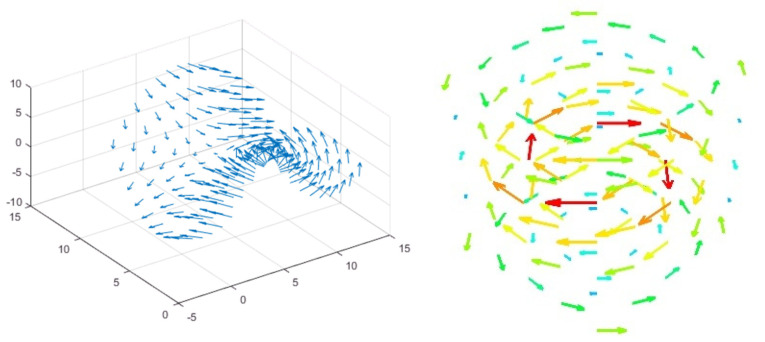
A 3D-representation of wind data using Matlab or Mayavi.

In future developments of the UAV-based mission planner module, we aim to incorporate a feature for exporting detailed 3D graphs, which will provide comprehensive insights into wind data across both horizontal and vertical dimensions. This enhancement will enable operators and researchers to visualize and analyze wind patterns in a multidimensional space, offering a far more granular understanding of the wind’s impact on maritime emission dispersion. The 3D graphs will plot wind speed and direction against geographic coordinates, creating a vivid representation of the wind’s behavior over the area of interest. To achieve this, we plan to utilize tools like Mayavi, a powerful 3D scientific data visualization library in Python, known for its ability to create dynamic and interactive 3D visualizations (Figure 7).

Such visualizations can be crucial for identifying patterns, trends, and anomalies in the wind data, which might otherwise be missed in traditional two-dimensional analyses.

### 7.6. Incorporating Edge Computing for Plume Dispersion Analysis

As we look towards future enhancements of the UAV-based mission planner module, a significant area of development is the integration of edge computing capabilities for advanced plume dispersion analysis. Edge computing, which involves processing data near the source rather than in a centralized cloud-based system, can significantly enhance the real-time analysis of environmental data. By integrating edge computing, the module could perform complex computations directly on the UAV mission control host, analyzing plume dispersion patterns in real-time. This capability would allow for the immediate generation of dispersion overlays on the mission map, providing operators with a more comprehensive and up-to-date understanding of the environmental situation.

The edge computing approach would enable a more sophisticated modeling of emission plumes, considering a variety of factors, such as wind speed, direction, atmospheric conditions, and topographical influences. With these detailed models, the UAV could generate and display predictive dispersion overlays, offering insights into the potential spread and impact of the emissions. This feature not only enhances the immediate operational effectiveness of the UAV but also contributes to long-term environmental monitoring and planning strategies. Such advancements in real-time data processing and visualization, enabled by edge computing, would mark a significant step forward in the capabilities of UAV-based environmental monitoring, providing invaluable tools for researchers and practitioners in the field.

## 8. Challenges and Limitations

The development of the UAV-based mission planner module for maritime emission monitoring, while innovative and promising, is not without its challenges and limitations. One of the primary challenges lies in the accuracy, reliability, and benchmarking [28] of wind sensing technology [17]. Despite significant advancements, real-time wind data collection in varying maritime conditions can be affected by factors such as sensor limitations, atmospheric interference, and the UAV’s own movement and vibrations. This can lead to discrepancies in wind measurement, impacting the module’s ability to provide precise data for emission monitoring.

Another notable challenge is the integration and synchronization of various technologies. The module relies on the seamless interaction between UAV hardware, wind sensors, the mission planner software, and real-time data processing algorithms. Ensuring consistent and error-free communication among these components, especially in a dynamic maritime environment, is complex and requires meticulous calibration and testing.

Furthermore, the reliance on SITL simulation for testing presents its own set of limitations. While SITL provides a controlled environment for initial testing and validation, it cannot fully replicate the unpredictability and complexity of real-world maritime conditions. This limitation underscores the need for extensive field testing to truly validate the module’s effectiveness in actual maritime emission monitoring scenarios.

There are also limitations related to the computational resources available on UAVs. The processing power required for real-time data analysis and mission path adjustment must be balanced with the UAV’s other operational demands, such as flight control and communication. This balance often necessitates trade-offs between computational capabilities and other critical functions.

Finally, regulatory and safety considerations pose additional challenges. The operation of UAVs, especially in sensitive or densely populated maritime areas, is subject to stringent regulations that may limit the scope of deployment. Ensuring compliance while maximizing the module’s effectiveness requires careful planning and consideration of legal frameworks.

Despite these challenges and limitations, this work represents a significant step forward in the use of UAV technology for environmental monitoring. By acknowledging and addressing these hurdles, further research and development can refine and enhance the module’s capabilities, paving the way for more effective and safer maritime emission monitoring.

## 9. Conclusions

The advancement of UAV technology for environmental monitoring, particularly in the challenging realm of maritime emission monitoring, represents a significant leap forward in our efforts to understand and mitigate the impacts of atmospheric pollution on coastal areas. The development and implementation of the mission planner module, as detailed in this paper, underscore the potential of integrating real-time wind sensing with UAV technology to enhance efficient and safe maritime emission monitoring. By harnessing the capabilities of Visual Studio 2022 and C# programming, this module not only succeeds in real-time processing and visualizing wind data but also introduces dynamic and user-friendly features to the GUI, significantly improving the operational experience for UAV operators.

The testing process, employing the SITL simulator and virtual machine setup, has proven the module’s ability to interpret wind data accurately and reliably, transforming this information into actionable insights through an intuitive and adaptive GUI. The enhancements made to the GUI, including dynamic arrows and color-coded text, along with the proposed future improvements, such as interactive data layers and advanced analytical tools, demonstrate our commitment to continuous innovation in this field.

This research significantly enhances the capabilities of environmental monitoring, introducing a tool that not only advances UAV mission control technology but also paves the way for more complex developments in this field. The project’s success inspires further exploration into sophisticated, collaborative monitoring systems, incorporating elements such as predictive analytics and augmented reality. These innovations are crucial in our fight against environmental degradation. Wind is a crucial factor for drone safety, and that’s why it is calculated and considered in risk assessment procedures. The impact of wind conditions on drone operations is significant, influencing flight stability, battery efficiency, and overall mission success. The importance of real-time wind prediction should not be underestimated, as wind has been a significant factor leading to potential loss or damage of drones. This is especially true in cases where drones lose battery power and are forced to land in the sea. By integrating wind data into risk assessments, operators can make more informed decisions, ensuring safer and more reliable drone missions. This aspect is vital for ensuring the longevity and reliability of UAVs in various environmental scenarios.

The proposed UAV-based module stands to revolutionize maritime emission monitoring, offering crucial benefits in plume path prediction and mission risk management. By accurately tracking emission plumes, it assists in anticipating environmental impacts and aids in strategic planning for emission control. Additionally, this technology significantly enhances operational safety. It enables operators to detect adverse wind conditions, thereby reducing the risk of equipment failure and ensuring failsafe landing protocols. This not only increases the reliability of maritime operations but also contributes to overall maritime safety and environmental responsibility. As we look ahead, the combination of real-time environmental data with these enhanced UAV systems shines as a beacon of hope, steering our efforts toward a more sustainable and environmentally conscious future.

## Figures and Tables

**Table 1:** MAVLink.MAVLINK_MSG_ID.WIND message.

Field Name	Units	Description
direction	deg (float)	Wind direction
speed	m/s (float)	Wind speed in ground plane
speed Z	m/s (float)	Vertical wind speed

**Table 2:** SITL wind parameters.

Field Name	Value	Description
SIM_WIND_DIR	120 deg	Simulated Wind Direction
SIM_WIND_SPEED	20 m/s	Simulated Wind Speed
SIM_WIND_TURB	15 m/s	Simulated Wind Variation
SIM_WIND_T_ALT	50 m	Full Wind Altitude

## Data Availability

In the spirit of collaboration and advancement in the field of maritime emission monitoring, we are pleased to announce that the complete source code for the mission planner module developed in this study is available on GitHub [29]. This repository includes all the relevant code files, documentation, and additional resources necessary for understanding and implementing the module. The code is shared under the usual usage rights associated with GitHub repositories, aiming to encourage further development, customization, and improvement by the wider scientific and developer community. By providing open access to our work, we hope to foster innovation and collective effort in enhancing UAV-based environmental monitoring technologies.
